# A Translational Murine Model of Sub-Lethal Intoxication with Shiga Toxin 2 Reveals Novel Ultrastructural Findings in the Brain Striatum

**DOI:** 10.1371/journal.pone.0055812

**Published:** 2013-01-31

**Authors:** Carla Tironi-Farinati, Patricia A. Geoghegan, Adriana Cangelosi, Alipio Pinto, C. Fabian Loidl, Jorge Goldstein

**Affiliations:** 1 Laboratorio de Neurofisiopatología, Departamento de Fisiología, Facultad de Medicina, Universidad de Buenos Aires, Ciudad Autónoma de Buenos Aires, Argentina; 2 Centro Nacional de Control de Calidad de Biológicos (CNCCB) – ANLIS “Dr. Carlos G. Malbrán”, Ciudad Autónoma de Buenos Aires, Argentina; 3 Instituto de Biología Celular y Neurociencia “Prof. E. De Robertis”, Facultad de Medicina, Universidad de Buenos Aires, Ciudad Autónoma de Buenos Aires, Argentina; St. Jude Children's Research Hospital, United States of America

## Abstract

Infection by Shiga toxin-producing *Escherichia coli* causes hemorrhagic colitis, hemolytic uremic syndrome (HUS), acute renal failure, and also central nervous system complications in around 30% of the children affected. Besides, neurological deficits are one of the most unrepairable and untreatable outcomes of HUS. Study of the striatum is relevant because basal ganglia are one of the brain areas most commonly affected in patients that have suffered from HUS and since the deleterious effects of a sub-lethal dose of Shiga toxin have never been studied in the striatum, the purpose of this study was to attempt to simulate an infection by Shiga toxin-producing *E. coli* in a murine model. To this end, intravenous administration of a sub-lethal dose of Shiga toxin 2 (0.5 ηg per mouse) was used and the correlation between neurological manifestations and ultrastructural changes in striatal brain cells was studied in detail. Neurological manifestations included significant motor behavior abnormalities in spontaneous motor activity, gait, pelvic elevation and hind limb activity eight days after administration of the toxin. Transmission electron microscopy revealed that the toxin caused early perivascular edema two days after administration, as well as significant damage in astrocytes four days after administration and significant damage in neurons and oligodendrocytes eight days after administration. Interrupted synapses and mast cell extravasation were also found eight days after administration of the toxin. We thus conclude that the chronological order of events observed in the striatum could explain the neurological disorders found eight days after administration of the toxin.

## Introduction

Infection by Shiga toxin (Stx)-producing enterohemorrhagic *Escherichia coli* (STEC) causes hemorrhagic colitis, hemolytic uremic syndrome (HUS) [1], acute renal failure [2], [3] and, less commonly, central nervous system (CNS) impairment [4]. In addition, it has been reported that the mortality rate from HUS increases from 0–5% to 7–40% when the CNS is involved [5]–[8]. This issue became prominent in Germany in 2011 when the consumption of sprouts containing Stx2 from the unusual enteroaggregative *E. coli* O104:H4 resulted in 3816 cases of gastroenteritis, 845 of which evolved to HUS and 54 to death. It was noteworthy that, although STEC strains usually develop HUS in children, in this case they affected adults, mainly women [9], [10]. In addition, 48% of the 217 hospitalized patients intoxicated with STEC in Germany developed severe neurological symptoms [11]. Argentina has the highest incidence of HUS in children under 5 years old (10.5 cases per 100,000) [12], [13]. Mortality and severe sequelae caused by STEC is still a persistent social and economic problem for international public health.

In children, neurological symptoms appear about eight days after the onset of hemorrhagic colitis. Symptoms include acute seizures, coma, irritability, hemiparesis, aphasia and motor alteration. Among these symptoms, seizures may potentially predict mortality or long-term neurological sequelae. It has been observed that neurological problems persist in 39% of surviving children [5]. Although the deleterious effects of STEC on the CNS are drastic, the pathogenic mechanisms whereby Stx2 causes CNS impairment still remain to be elucidated.

Several animal models have been used to study the effects of Stx on the CNS [14]–[18]. However, none has studied the deleterious effects of intravenous administration of Stx2 on the striatum. The neurological alterations described among models of Stx2 injection in mice include hind limb paralysis [19], lethargy, shivering, abnormal gait and spasm-like seizures [20]. Vascular and glial changes have been observed at light microscope level [19], [21], [22]. Intraperitoneal administration of Stx2 causes both a glial lamellipodia-like process that blocks spinal motor neuron synapses [20] and endothelial damage in the cerebral cortex, hippocampus and cerebellum [16]–[18], [23], [24]. In the rat model of intracerebroventricular (i.c.v.) Stx2 administration, ultrastructural observation has revealed an apoptotic form of neuronal degeneration, demyelination, astrogliosis and pathological oligodendrocytes [25], [26]. Rabbit models of Stx injection have shown endothelial alterations like edema and hemorrhage [27]–[29], as well as alterations in the myelin sheath, neuronal degeneration, gliosis, vascular changes and hemorrhage in different brain areas [30]–[32]. Pigs have been found to develop edema disease characterized by CNS symptoms such as ataxia, and incoordination [33], convulsions, leg paddling and paralysis [34]–[37]. In baboons, seizure episodes which progressed to coma and death after i.v. administration of Stx2 have also been reported [38]. Stx1 causes ultrastructural lesions that are not observable with light microscopy [39]. More details of animal models of Stx administration can be found in a review written by Obata (2010) [40].

The study of the striatum is relevant because basal ganglia are one of the most common brain areas affected in patients that have suffered from HUS [41]–[43]. The striatum modulates the central motor system [44], [45] and is one of the most relevant brain regions involved in neurological motor disorders in patients intoxicated with STEC [46], [47]. Magnetic resonance images (MRIs) have revealed that basal ganglia are frequently involved in this pathology [42], [48]. A correlation between neurological deficits and radiological images related to basal ganglia compromise in comatose patients has been frequently reported [46], [47]. However, non-invasive methods such as MRI may not identify which cell type is damaged or the extent of cell damage over a certain period of the known toxin progression. In contrast, transmission electron microscopy (TEM) is a powerful technique to detect the ultrastructural state of these cells.

Thus, since the effects of sub-lethal i.v. doses of Stx2, particularly in the striatum, have never been studied in an animal model, in the present study we attempted to determine the type of ultrastructural changes produced by Stx in the striatum and to what extent these changes may resemble the human disease. We also describe the correlation between the neurological alterations observed and blood brain barrier disruption, mast cell extravasation, synapse interruption and striatal cell death.

## Materials and Methods

### Stx2 Purification

Stx2 was obtained as previously described [26]. Briefly, the toxin was purified by affinity chromatography under native conditions. Recombinant *E. coli* DH5α containing pStx2 were cultured overnight. The supernatant obtained was precipitated in 60% SO_4_ (NH4)_2_ 1 mM PMSF, and the pellet was dialyzed overnight, resuspended in phosphate buffer solution (PBS) with a cocktail of protease inhibitors, and incubated with Globotriose Fractogel Resin (IsoSep AB, Tullinge, Sweden). The resin was washed and the toxin eluted with MgCl_2_. Protein concentration was determined in all the eluates. Protein content in all the fractions was monitored by silver/Coomassie blue staining [49], and the presence of Stx2 in the eluates was confirmed by Western Blot analysis. Results showed a 7.7-kDa band corresponding to Stx2B and a 32-kDa band corresponding to Stx2A. The same batch of toxin was used for all the experiments.

The cytotoxic capacity of Stx2 was assessed in Vero cells by the neutral red assay and the cytotoxic dose 50 (CD_50_) found was about 1 pg/ml [50]. This effect was neutralized by means of preincubation with an anti-subunit 2B monoclonal antibody (Sifin, Berlin, Germany), and not neutralized when using an isotype antibody instead [50]. Lipopolysaccharide (LPS) was removed from the Stx2 solution by using Detoxi-gel (Pierce, Rockford, USA). This Stx2 solution contained less than 0.03 endotoxin units/ml.

### Sub-lethal dose

The lethal effects of purified Stx2 were characterized in mice (n = 4 per dose). Different amounts of Stx2 (5 to 0.44 ηg per animal) or vehicle were administered intravenously (i.v.) to mice weighing about 20 grams. Survival time was established when 100% of the animals survived at least 8 days after administration of 0.5 ηg or less of Stx2. It was determined that with this dose mice survived even for 10 days. This amount was thus considered sub-lethal and selected for use in this study. The toxin was administered twice (once a day for two consecutive days) with time zero prior to initial administration of the toxin.

### Animals

Male NIH mice (28–30 days old) were housed in an air-conditioned and light-controlled (lights on between 7:00 am and 7:00 pm) animal facility. Animals were supplied by the animal facility center of Administración Nacional de Laboratorios e Institutos de la Salud (ANLIS) Carlos G. Malbrán, Buenos Aires, Argentina. Mice were provided with food and water *ad libitum* and neurological manifestations were monitored daily at the same time throughout the experiment. The experimental protocols and euthanasia procedures were reviewed and approved by the Institutional Animal Care and Use Committee of the School of Medicine of Universidad de Buenos Aires, Argentina (Resolution No. 1099/10). All the procedures were performed in accordance with the EEC guidelines for care and use of experimental animals (EEC Council 86/609).

### Behavioral motor tests

Mice were subjected to motor behavioral tests based on a primary screening using a modified version of the SHIRPA-test [51], [52] 2, 4 and 8 days after i.v. administration of Stx2 or vehicle. For the purpose of clarity, non-motor SHIRPA-screen behavioral tests were not included. A total of three experiments were performed using 12 mice per treatment. Animals were placed in individual cages and the following motor behaviors were recorded over a 5-minute period, without disturbing the animal: spontaneous motor activity (i.e. whether the animals had vigorous, moderate, slow or no activity at all), gait (i.e. normal or abnormal), pelvic elevation (i.e. whether animals were able to elevate their body more than 3 mm from their horizontal axis, either normal (not deviated from the axis) or barely touching the ground), and hind limb activity (i.e. whether animals had normal or limited hind limb movements). All the animals subjected to the treatments described were given a score based on their motor capacity in the motor tests. Normal behavior (vigorous activity, normal correct posture, body not deviated from the axis and fluid hind limb activity) was given a score of 0 and abnormal behaviors were given a score of 1. The formula to calculate the SHIRPA score is:
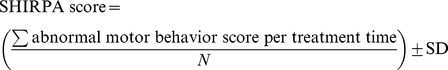



where n (number of tests) = 4. Table 1 lists the motor behavioral tests that showed significant differences between vehicle- and Stx2-treated animals.

**Table 1:** Behavioral motor tests.

Test	Score = 0	Score = 1
Spontaneous motor activity	Vigorous movement	Slow movement
Gait	Normal	Abnormal
Pelvic Elevation	Normal (elevation of 3 mm)	Elevated (elevation of more than 3 mm)
Hind limb activity	Normal	Difficulty to move

### Transmission Electronic Microscopy (TEM)

Mice were anesthetized with Chloral hydrate (350 mg/kg) and perfused transcardially with 0.9% NaCl solution followed by 2.5% glutaraldehyde in 0.1 M PBS [fixative per animal/weight (ml/g)] for studies of Transmission Electron Microscopy (TEM). Brains were removed from the skull and post-fixed in the same fixative solution for 2 hours. Samples of dorsal striatum (3 mm^2^ thick) were dissected and collected in 0.1 M phosphate buffer. The samples were first assessed by light microscopy with blue toluidine to select the areas for TEM. Ultrathin sections were cut from selected areas [53] and then contrasted with 1% OsO4 and 1% uranyl acetate, dehydrated and flat-embedded in Durcupan. The sections were contrasted with lead citrate and then examined and photographed on a Zeiss 109 electron microscope. Adobe Photoshop software was used in the assembly of images (Adobe Systems Inc., San Jose, CA, USA).

### Statistical analysis

Twelve animals from each treatment group were subjected to behavioral motor test studies. Four brain sections from each of four animals (i.e. a total of 16 samples for each condition) were used for TEM studies. The data are presented as mean ± SEM. Statistical significance was assessed by Student-t-test or one-way analysis of variance (ANOVA) followed by Bonferroni post tests (GraphPad Prism 4, GraphPad Software, Inc.). P values <0.05 were considered significant.

## Results

**Figure 1:**
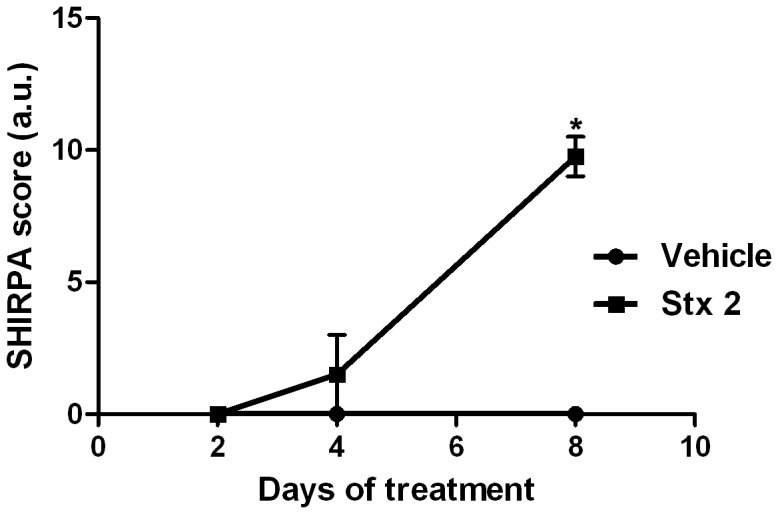
Behavioral motor test. Plotting of SHIRPA scores to standardize results of four motor behavioral tests (spontaneous motor activity, gait, pelvic elevation and hind limb activity). Arbitrary units (a.u.) are the mean summation of the four independent tests for each day and treatment (vehicle or Stx2). Normal behavior = 0; abnormal behavior = 1. The vehicle-treated group (vehicle) shows no abnormal behavior while the Stx2-treated group (Stx2) shows significant abnormal behavior (*) on day 8 of toxin treatment (p<0.05).

To test whether sub-lethal i.v. administration of Stx2 altered motor behavioral function in mice, motor behavioral tests consisting of four neurological assessments –spontaneous motor activity, gait test, pelvic elevation and hind limb activity– were conducted 2, 4 and 8 days after administration (Fig. 1). Results evidenced motor functional deficiencies in which striatal damage could be involved. Spontaneous motor activity of Stx2-treated mice was reduced by 50% 4 days after administration and by 100% 8 days after administration. Reduced hind limb activity, abnormal gait and increased pelvic elevation were observed in 75% of the Stx2-treated mice 8 days after administration. No neurological differences were found between Stx2-treated mice and the vehicle-injected group 2 days after administration. Spontaneous motor activity, gait test, pelvic elevation and hind limb activity were normal in vehicle-treated mice throughout the eight experimental days. Behavioral changes in sensorial and learning tasks were also monitored (data not shown). Consequently, a detailed ultrastructural study was performed.

### Intravenous administration of Stx2 causes necrotic and degenerative-like neurons

**Figure 2:**
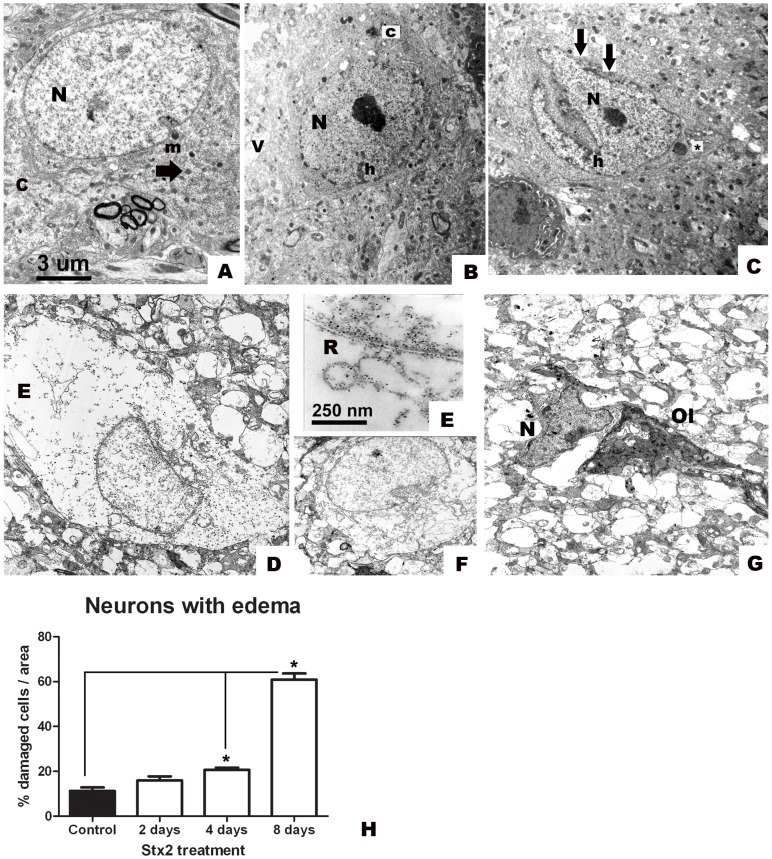
Intravenous administration of Stx2 causes neuronal damage. Conserved striatal neuron after i.v. vehicle (saline) administration (A); pale nucleus (N) and intact cytoplasm (c) and membranes (m); intact mitochondrion (arrow). After 2 days of treatment: vacuolated neuron with contrasted cytoplasm (c) and nucleus (N) (B); heterochromatin condensation (h). After 4 days: more contrasted nucleus (N) with increased heterochromatin (h) condensation and prominent indentation with loss of membranes (arrows) (C); vacuolated perinuclear mitochondrion (*) (C). Neuron with edema and loss of regular nuclear shape on day 8 (D). Disorganized endoplasmic reticulum (R) in the cytoplasm with edema (E) at higher magnification; loss of regular shape in the nucleus (F) of a neuron with edema (E); affected neuronal nucleus (N) with irregular shape and no apparent surrounded cytoplasm; (Ol, oligodendrocyte) (G). These features are absent in striatal neurons of the vehicle-treated group (A). Quantification of the percentage of damaged neurons with edema (H). Significant results are observed starting on day 4. Maximum number of neurons with edema in Stx2-treated mice observed on day 8 (*) (H). Results are expressed as a percentage of the total number of neurons in an area of 3721 µm^2^. Data are mean ±SEM of 6–8 experiments (F and G). An asterisk denotes statistical significance, p<0.05. The scale bar in A applies to micrographs B–D and F–G.

Conserved neurons of the striatum from the vehicle-treated group showed pale nuclei and well-dispersed chromatin, intact cytoplasmic and nuclear membranes, and intact mitochondria dispersed in the cytoplasm (Fig. 2A). In contrast, striatal neurons from the Stx2-treated group showed a progressive degenerative condition (Fig. 2B–F). Two days after administration, the cytoplasm started to become vacuolated and more contrasted than in the vehicle-injected group (2B). Two and four days after administration, nuclei in the samples from Stx2-treated mice appeared electron dense, with increased heterochromatin condensation, and resembled the early changes seen in an apoptotic state (2B, C). In these neurons, perinuclear clustering of altered mitochondria occurred within darkened and condensed cytoplasm. In addition, altered nuclear and cytoplasmic membranes were observed by ultrastructure two days after administration. Loss and/or convergence of the two membranes was observed as an earlier sign of neuronal damage. This feature could be seen only by TEM. Indentations of the nuclear membrane were frequently found 4 days after administration, compared with the nuclei from the vehicle-treated group (Fig. 2C). The changes observed over this period reflected a pathological condition as nuclear indentation would be consistent with the beginning of an apoptotic process. Eight days after administration, the predominant ultrastructural observation included neurons with cytoplasmic edema and shrunk nuclei (Fig. 2D, F), or neurons that were already in a necrotic state (Fig. 2G). Complete alteration of the endoplasmic reticulum, organelles and mitochondria were observed in neurons with edema (Fig. 2B, C). A common feature observed over the time periods analyzed was cell edema, which increased over time (Fig. 2H) and was significantly maximum (p<0.05) 8 days after Stx administration.

### Intravenous administration of Stx2 causes edema in astrocytes and mast cell infiltration

**Figure 3:**
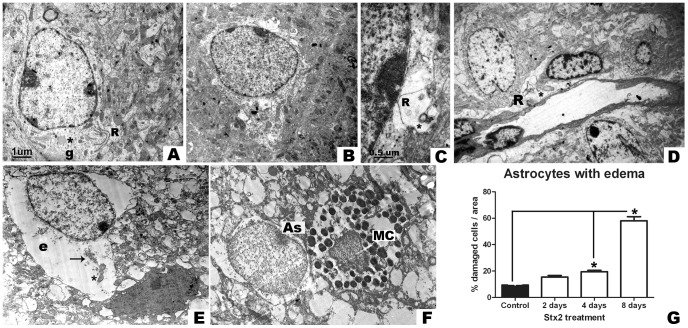
Intravenous administration of Stx2 causes astrocytic edema and mast cell extravasation. Electron micrograph showing a conserved astrocyte after i.v. vehicle administration (A); conserved mitochondria (*), endoplasmic reticulum (R) and gliofilaments (g). After 2 days: astrocytes show significant cytoplasmic edema (B); disorganized endoplasmic reticulum (R) and swollen mitochondria (*) (C). After 4 days: evident astrocytic edema along the perivasculature (D); disorganized endoplasmic reticulum (R) and swollen mitochondria (*) with disorganized inner membranes. After 8 days: altered astrocyte showing watery cytoplasmic edema (e), disorganized rough endoplasmic reticulum (arrow) and mitochondria with broken membranes (*) (E); a neighboring conserved oligodendrocyte can be observed. Extravasation of a mast cell (observable granules, MC) in contact with an astrocyte (As) (F). Quantification of the percentage of damaged astrocytes showing edema (G). Significant results are observed starting on day 4. The maximum number of astrocytes with edema in Stx2-treated mice is observed on day 8 (*). Results are expressed as a percentage of the total number of astrocytes in an area of 3721 µm^2^. Data are mean ±SEM of 6–8 experiments (G). An asterisk denotes statistical significance, p<0.05. The scale bar in A applies to A, B, and D–F electron micrographs.

Normal astrocytes were observed in samples from vehicle-treated mice, usually together with mitochondria, rough endoplasmic reticulum, Golgi apparatus, dispersed small vesicles and glial filaments (Fig. 3A). In contrast, altered astrocytes were observed in Stx2-treated mice after day 2 (Fig. 3B). About 20% of the astrocytes observed exhibited edema (Fig. 3G). This was accompanied by evident disorganized endoplasmic reticulum and swollen mitochondria. However, the nuclear envelope was conserved (Fig. 3C). On day 4, perivascular astrocytic edema was also observed (Fig. 3D). Disorganized endomembranes and swollen mitochondria were found in watery cytoplasm. In contrast, nuclear integrity was not compromised (Fig. 3D). On day 8, loss of nuclear electron dense elements that migrated to the cytosol probably due to nuclear membrane rupture and swollen organelles in watery cytoplasm was evidenced (Fig. 3E). Cytoplasmic edema was the main feature observed throughout the experiment (Figs. 3B–F). The percentage of astrocytes that displayed edema increased to 60% on day 8 and was significantly higher than in vehicle- or Stx2-treated mice after 2 or 4 days (Fig. 3G). Stx2 caused astrocyte damage in the brain striatum. In addition, mast cell extravasation to the brain parenchyma was likely (Fig. 3F). Mast cells are identified by ultrastructure because they have numerous rounded non-laminated granules in comparison with basophil cells, which have larger and laminated granules. Mast cells may go through the Blood Brain Barrier (BBB) into the brain parenchyma attracted by inflammatory mechanisms after the Stx2 insult. Here, a mast cell in contact with an astrocyte was observed (Fig. 3C). Mast cells are early responders in the regulation of acute BBB changes after cerebral ischemia and hemorrhage.

### Intravenous administration of Stx2 alters BBB integrity

**Figure 4:**
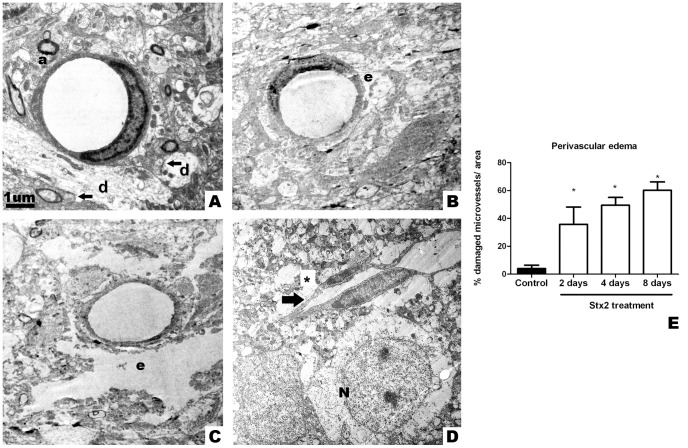
Intravenous administration of Stx2 causes ultrastructural alterations at the blood brain barrier level. Electron micrograph showing a conserved endothelial cell with conserved endothelial nucleus that constitutes a microvessel from a striatal brain slice after i.v. administration of vehicle; microvessel surrounded by conserved synapses (arrows), dendrites (d) and myelinated axons (a) (A). Perivascular edema (e) 2 days after i.v. administration of Stx2 (B). After 4 days: more pronounced perivascular edema (e) (C). After 8 days: infarcted microvessel (arrow) with perivascular edema (*) near a neuron (N) (D). No cytoplasmic membrane is observed in the neuron at this stage. Quantification of the percentage of damaged microvessels by perivascular edema (E). Damage in microvessels begins to be significant on day 2 and is maximum on day 8 (*). Results are expressed as a percentage of the total number of microvessels in an area of 3721 µm^2^. Data are mean ±SEM of 6–8 experiments (G). An asterisk denotes statistical significance, p<0.05. The scale bar in A applies to micrographs B–D.

Endothelial vessels from striatal brain sections of vehicle-injected mice kept their endothelial nucleus and cytoplasm conserved (Fig. 4A). Organelles were well preserved and mitochondria showed normal external and inner membranes. Since the vascular basal membrane was found covered by intact perivascular astrocytes, the BBB appeared intact. Intact perivascular astrocytes contacted dendrites and axons covered with a sheath of myelin. In contrast, a progressive intracytoplasmic edema of perivascular astrocytes was observed after i.v. administration of Stx2 starting on day 2 (Fig. 4B). An incipient edema was evidenced in perivascular astrocytes on day 2, while a complete watery edema was observed mainly on day 4 (Fig. 4C). Watery edema with collapsed and shrunk endothelial cells was observed on day 8 (Fig. 4D), although no apparent endothelial cell damage was observed. Complete watery edema was maximum on day 8 (Fig. 4E). Accordingly, perivascular edema increased over time.

### Intravenous administration of Stx2 causes interrupted synapses

**Figure 5:**
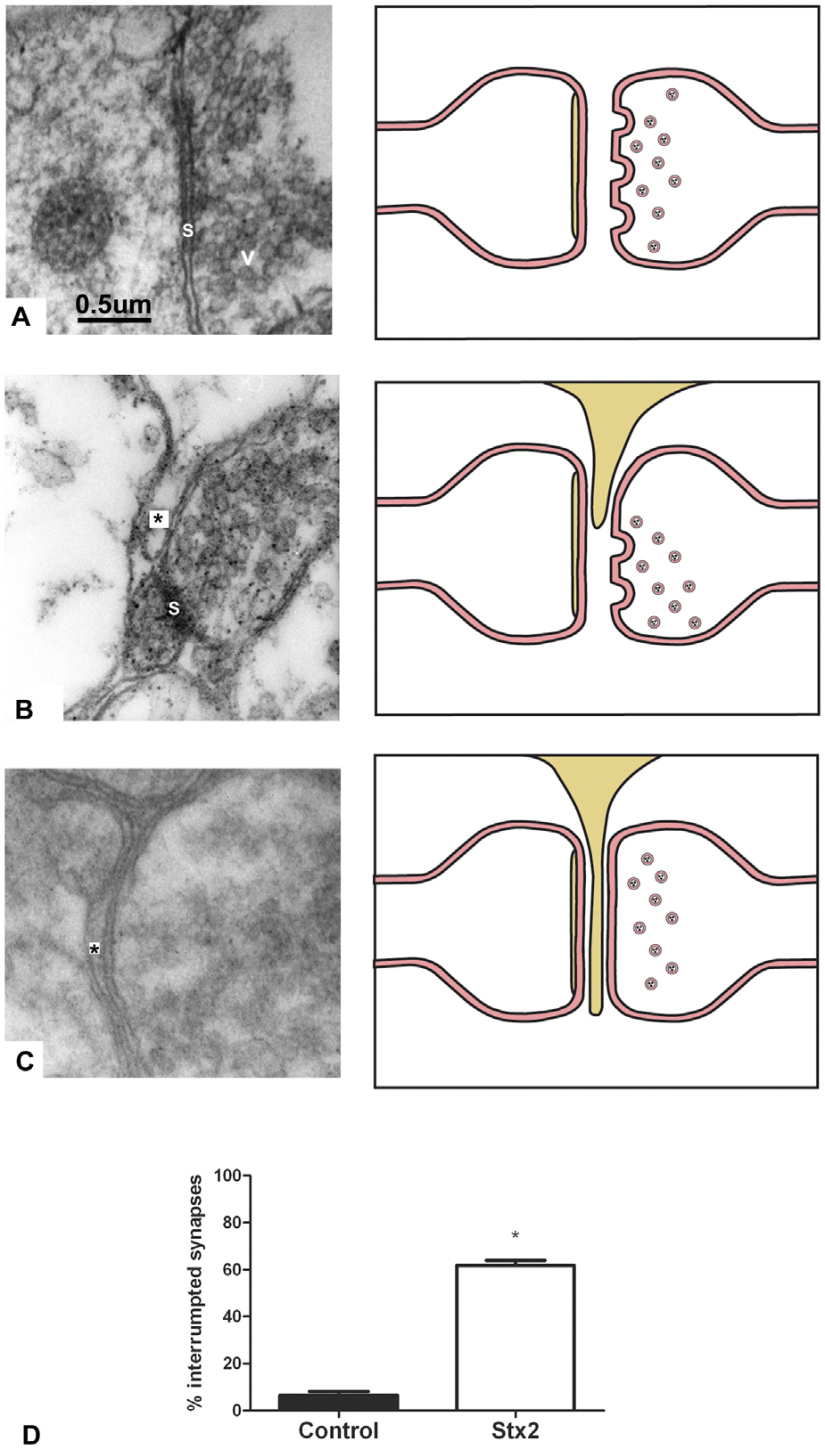
Intravenous administration of Stx2 causes synaptic modifications. Electron micrograph showing an apparently normal synapse (s) and pre-synaptic vesicles (v) in the striatum (A). Electron micrograph showing an increased number of pre-synaptic vesicles together with an incipient astrocytic process (*) in close proximity to a synaptic space (S) in the lower left corner (B). Electron micrograph showing full astrocytic interposition in the synaptic space (*) while the pre-synaptic terminal is full of vesicles (C). Chart illustrating the process in the synaptic space (right panel). Percentage of interrupted synapses in the striatum from Stx2-treated mice vs. vehicle-treated ones (D). An asterisk denotes statistical significance, p<0.05. Results are expressed as a percentage of the total number of synapses in an area of 3721 µm^2^. The scale bar in A applies to all micrographs.

Interrupted synapses were observed in the striatum of Stx-2-treated mice (Fig. 5). The number of interrupted synapses was significantly higher in Stx2-treated animals than in vehicle-treated ones. A projection of a reactive astrocyte interrupted the synaptic transmission of neurons by standing between the pre- and the post-synapse (Fig. 5B, C).

### Intravenous administration of Stx2 causes ultrastructural alterations in oligodendrocytes

**Figure 6:**
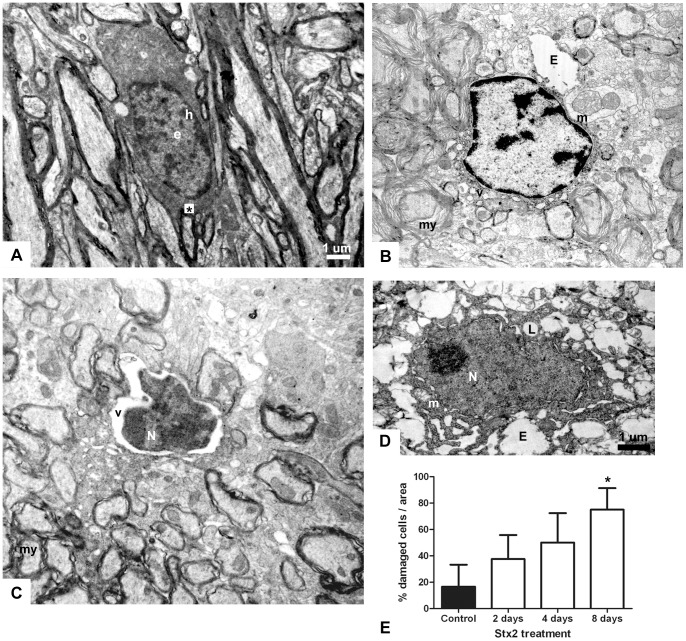
Intravenous administration of Stx2 causes pathological alterations in oligodendrocytes. Conserved oligodendrocyte in striata of brains treated with i.v. administration of vehicle; myelin formation in the cytosol (*) (A); electron-dense heterochromatin (h) mostly in the periphery of a rounded nucleus; euchromatin (e) present as electron-lucent areas of the inner nucleus. An irregular nucleus is observed 2 days after Stx2 treatment (B): vacuolated cytoplasm (v); disorganized myelin (my); Edema (E) and swollen mitochondria in the extracellular space. A shrunk and retracted nucleus (N) and perinuclear vacuolization (v) are observed on day 4 (C). Altered oligodendrocyte with a contrasted cytoplasm observed on day 8 (D); nucleus (N) and cytoplasm are difficult to differentiate; extended edema (E) and lack of myelin (L); swollen and disorganized cristae in mitochondria (m). The number of cells with edema increased with time (E). Results are expressed as a percentage of the total number of oligodendrocytes in an area of 3721 µm^2^. Data are mean ±SEM of 6–8 experiments. An asterisk denotes statistical significance, p<0.05. The scale bar in A applies to B and C.

Normal oligodendrocytes were identified by the presence of round or oval nuclei containing heterochromatin in the periphery of the nucleus and electron-dense dark cytoplasm in vehicle-treated mice (Fig. 6A). Oligodendrocytes were also distinguished for their cytoplasm ensheathed axons. Two days after i.v. administration of Stx2, nuclei of affected oligodendrocytes started to show an irregular shape, condensed chromatin and mild retraction (Fig. 6B). However, the nuclear envelope was still intact. In the perinucleus, an electron-dense cytoplasm with partial vacuolization was evident. The ultrastructural features observed suggest an early stage of apoptosis. In addition, disorganized myelin sheaths were observed in apparently swollen axons. On day 4, a progressive deterioration of the oligodendrocytes was observed (Fig. 6C). Nuclei became more irregular and shrunk. Further retraction of the nucleus was observed and bulked chromatin was evident. Nuclear fragments were also observed in the edematous area. At this stage, perinuclear vacuolization extended to the whole electron-dense cytoplasm and myelin appeared to be not only disorganized but also thinner than on day 2. Axons covered with myelin were irregular and electron dense contrasted. On day 8, oligodendrocytes showed contrasted nuclei and cytoplasm, and boundaries between them were difficult to differentiate (Fig. 6D). Loss of cell volume immersed in an edematous area was the main feature at this stage. However, cytoplasmic projections that resembled sheaths covering axons were still observable. Cell damage, quantified on days 2, 4, and 8 according to the criteria previously described (Fig. 6E), increased over time. Accordingly, the number of cells displaying damage was significantly higher on day 8 than on days 4 and 2.

## Discussion

Sub-lethal administration of Stx2 caused motor behavioral alterations that may be related to damage of striatal cells. To our knowledge, this is the first report on the toxic effects of sub-lethal doses of i.v. administration of Stx2 in mouse brain striatum.

The striatum is part of the basal ganglia, with a relevant role in the control of voluntary movements and in the onset of disorders [44], [45]. This brain area is highly affected in patients intoxicated with STEC [42], [43], [46]–[48]. Neurological complications have been found in patients with HUS and basal ganglia injury after 6–7 days of the onset of symptoms [47]. Although hind limb paralysis has been described in animal models as a disturbance caused by Stx2, this deficit has been related only to spinal cord motor neurons and neither this nor other motor deficits have been associated with any particular brain region [19], [20]. The neurological deficits found in this study on the basis of motor behavioral tests could be correlated with the damage observed in the striatum and are consistent with the mean time of onset of symptoms in patients that suffer from neurological manifestation of HUS [5]. Other brain regions associated with movement, including the motor cortex and the pyramidal tract, may also be implicated in damage by Stx2.

Neurons from vehicle-treated striatum showed a normal appearance compared with Stx2-treated ones [54], [55]. In contrast, ultrastructural alterations observed in the striatum after intravenous Stx2 administration showed earlier signs of neuronal damage and an apoptotic state [54], [56], [57]. Pathological nuclear indentations frequently found in neurons on day 4 have also been found in Huntington's chorea disease neurons in related animal models [58]–[60] or following axonal injury [61]. Overall, the altered changes observed in these neurons reflect a pathological condition of Stx2-treated mice.

In accordance with previous reports, normal astrocytes were observed in vehicle treated-mice [62]. Astrocytes usually cover the basement membrane of pial and vascular surfaces in the CNS, and they surround neuronal bodies, axons and other neuronal processes within the CNS. Under pathological conditions, astrocytes may show increase in volume and large quantities of filaments or watery swelling [62], [63]. Failure of functionality in the nuclear envelope could be the consequence of the osmotic changes caused by the edema.

The microvasculature in the striatum from vehicle-treated mice showed a conserved BBB ultrastructure [64], [65] as compared with that of Stx2-treated ones. Fujii et al. [14] found edematous endothelial cells in the cortex of a moribund mouse 2 days after injection of a lethal dose of Stx2 (4 ηg per mouse). In contrast, we have shown that a sub-lethal dose of 0.5 ηg per mouse was sufficient to cause a typical perivascular edema [64] and BBB disruption by altering perivascular astrocytes. While we have previously shown that intracerebral Stx2 causes vascular leakage [26], in the present study we found that the toxin disrupts the BBB.

Interposition of astrocytic processes between the pre- and post-synaptic terminals, namely astrocytic stripping, was observed in the striatum. A similar physical blocking of lamellipodia-like processes in the synapses of mouse motoneurons following Stx2 treatment has been described [20]. Synapse interruption could be a neuroprotective mechanism whereby projections from astrocytes may physically detach the synapse against excessive pre-synaptic release of glutamate [66], [67]. The altered synaptic terminals may be the consequence of excessive glutamate release [20], [68]. Therefore, when an astrocytic interposition between a pre- and a post-synaptic terminal occurs, post-synaptic densities may not be observed.

Mast cells were identified [69] and localized in the parenchymal striatum, probably due to the pathological condition caused by the toxin. These cells are usually located in the perivasculature [70] and migrate into the brain parenchyma by an ischemic event following brain swelling or alteration of BBB permeability [71]. Peripheral factors produced by Stx2 might contribute to mast cell infiltration and astrocytic stripping, as these findings have not been observed after local administration of Stx2 in rat striatum [26]. Nevertheless, structural differences between species might account for this difference.

The oligodendrocytic phenotype may still be observed even in the pathological condition [72]. In addition, oligodendrocytes and microglial cells might be erroneously identified, unless ultrastructural differences become evident. When microglial cells become activated by a noxious agent they display dilation of the endoplasmic reticulum channels and a great number of electron light vesicles in the cytoplasm. Also, a small number of activated cells have electron-dense lysosomes, indicating phagocytic activity [73]. As these features were not found in the injured cells, they corresponded to oligodendrocytes. Apoptosis in oligodendrocytes was observed at early stages [72], while necrosis was observed at later stages [72], [74]. Damage in oligondendrocytes might cause the axonal transmission deficits observed in Stx2-treated mice, with motor alterations including hind limb paralysis. Demyelinated axons have been observed after the intracerebroventricular administration of Stx2 [26].

Stx2 has been immunolocalized in the brain parenchyma [20], [26]. Although the receptor for Stx2 Gb3 is not localized in the mouse brain endothelium [20], the toxin is nevertheless able to bind to neurons. However, the mechanism whereby Stx2 enters the brain parenchyma is still unknown. The BBB must keep a healthy fluid environment to protect the brain parenchyma [13], [65] from harmful elements and to allow homeostatic influx of various required substances and efflux of cell products to maintain CNS homeostasis [71], [75] and our results show that vascular leakage as a result of Stx2 would alter this homeostasis. The toxin may cross the brain parenchyma through vessels of circumventricular organs that lack tight junctions, or by destroying them in the non-fenestrated endothelia, or by first crossing the blood-cerebrospinal fluid barrier to the ventricle space and then to the brain parenchyma by crossing ependymal cells. Another possibility is that the toxin crosses the BBB as a result of damage of perivascular astrocytes that constitute a part of the barrier (Fig. 4).

The possible mechanisms behind the cell damage observed include: i) cytotoxic action of Stx2 mediated by the Gb3 receptor in neurons [20], [50]; ii) contribution of peripheral or locally produced pro-inflammatory cytokines and/or chemokines [76]; iii) deleterious collateral effect produced by kidney failure, which could lead to electrolyte disorders and target the brain [76]; and iv) energy depletion by lack of glucose and oxygen intake.

Regarding the second possible mechanism, it has been widely reported that TNF-α contributes to the deleterious effects of Stx2 both in vitro [77] and in vivo [78]. Also, cytokines produced in the brain have been reported [32] and their inflammatory effect could partially explain the astroglial reactivity observed [25]. Under this circumstance they can release more toxic products and target cells in the vicinity, including neurons [79].

With regard to the fourth possible mechanism, BBB rupture could prevent the incorporation of these substrates to neurons and other cells. As a consequence, energy depletion would cause membrane depolarization and the release of excitatory amino acids into the extracellular space, leading to edema and apoptosis in neurons and astrocytes [79].

In the present study, the ultrastructural lesions observed in the striatal cells could have caused the motor behavioral alterations found in Stx-treated animals. However, possible lesions produced in other brain centers like the motor cortex and the pyramidal tract cannot be discarded. The main input structure of the basal ganglia is the striatum [80], which is specifically related to voluntary limb movements [81]. The mechanistic entry of Stx2 to the brain parenchyma and the subsequent events that lead to neuronal and glial degenerative conditions are a current matter of study. Other factors besides Stx2 may contribute to the described pathology, and they should also be taken into account.

In summary, a sub-lethal i.v. dose of Stx2 caused BBB disruption, astrocytic edema, neurodegeneration, oligodendrocyte death, synapse interruption and mast cell extravasation, which may explain the motor behavioral alterations and lethargy observed in mice. This damage may also reflect some clinical alterations observed in brain impairment by the toxin. Therefore, this animal model appears to be useful to compare and understand the basis of the neuropathogenic mechanisms underlying STEC intoxication in patients.

## References

[1] O'Brien AD, Kaper JB (1998) Shiga toxin-producing Escherichia coli: yesterday, today, and tomorrow. In: Kaper JB, O'Brien AD (Eds). Escherichia coli O157:H7 and Other Shiga Toxin-Producing E. coli Strains. Am Soc Microbiology Washington DC, pp. 1–11.

[2] Proulx F, Seidman EG, Karpman D (2001) Pathogenesis of Shiga toxin-associated hemolytic uremic syndrome. Pediatr Res 50: 163–171. 11477199 10.1203/00006450-200108000-00002

[3] Karmali MA (2004) Infection by Shiga toxin-producing Escherichia coli: an overview. Mol Biotechnol 26: 117–122. 14764937 10.1385/MB:26:2:117

[4] Cimolai N, Carter JE (1998) Bacterial genotype and neurological complications of *Escherichia coli* O157:H7-associated haemolytic uraemic syndrome. ActaPædiatr 87: 593–594. 10.1080/08035259850158353 9641746

[5] Eriksson KJ, Boyd SG, Tasker RC (2001) Acute neurology and neurophysiology of haemolytic-uraemic syndrome. Arch Dis Child 84: 434–435. 11316694 10.1136/adc.84.5.434 PMC1718775

[6] Upadhyaya K, Barwick K, Fishaut M, Kashgarian M, Siegel NJ (1980) The importance of nonrenal involvement in hemolytic uremic syndrome. Pediatrics 65: 115–120. 7355005

[7] Shetb KJ, Swick HM, Haworth N (1986) Neurologic involvement in hemolytic uremic syndrome. Ann Neurol 19: 90–99. 3947042 10.1002/ana.410190120

[8] Hahn JS, Havens PL, Higgins JJ, O'Rourke PP, Estroff JA, et al (1989) Neurologic complications of hemolytic uremic syndrome. J Child Neurol 4: 108–113. 2715605 10.1177/088307388900400206

[9] Struelens MJ, Palm D, Takkinen J (2011) Enteroaggregative, Shiga toxin producing *Escherichia coli* O104:H4 outbreak: new microbiological findings boost coordinated investigations by European public health laboratories. Euro Surveill 16: pii = 19890 10.2807/ese.16.24.19890-en 21699771

[10] Frank C, Werber D, Cramer JP, Askar M, Faber M, et al (2011) Epidemic profile of Shiga-toxin-producing Escherichia coli O104:H4 outbreak in Germany. N Engl J Med. 365: 1771–1780. 10.1056/NEJMoa1106483 21696328

[11] Magnus T, Röther J, Simova O, Meier-Cillien M, Repenthin J, et al (2012) The neurological syndrome in adults during the 2011 northern German E. coli serotype O104:H4 outbreak. Brain 135: 1850–1859. 22539260 10.1093/brain/aws090

[12] Noris M, Remuzzi G (2005) Hemolytic uremic syndrome. J Am SocNephrol 16: 1035–1050. 10.1681/ASN.2004100861 15728781

[13] Meichtri L, Miliwebsky E, Gioffré A, Chinen I, Baschkier A, et al (2004) Shiga toxin-producing Escherichia coli in healthy young beef steers from Argentina: prevalence and virulence properties. Int J Food Microbiol. 96: 189–198. 10.1016/j.ijfoodmicro.2004.03.018 15364473

[14] Fujii J, Kita T, Yoshida S, Takeda T, Kobayashi H, et al (1994) Direct evidence of neuron impairment by oral infection with verotoxin-producing *Escherichia coli* O157:H- in mitomycin-treated mice. Infect Immun 62: 3447–3453. 8039916 10.1128/iai.62.8.3447-3453.1994 PMC302977

[15] Karpman D, Connell H, Svensson M, Scheutz F, Alm P, et al (1997) The role of lipopolysaccharide and Shiga-like toxin in a mouse model of Escherichia coli O157: H7 infection. J Infect Dis 175: 611–620. 9041333 10.1093/infdis/175.3.611

[16] Kurioka T, Yunou Y, Kita E (1998) Enhancement of susceptibility to Shiga toxin producing Escherichia coli O157:H7 by protein calorie malnutrition in mice. Infect Immun 66: 1726–1734. 9529103 10.1128/iai.66.4.1726-1734.1998 PMC108110

[17] Isogai E, Isogai H, Kimura K, Hayashi S, Kubota T, et al (1998) Role of tumor necrosis factor alpha in gnotobiotic mice infected with an Escherichia coli O157:H7 strain. Infect Immun 66: 197–202. 9423858 10.1128/iai.66.1.197-202.1998 PMC107877

[18] Taguchi H, Takahashi M, Yamaguchi H, Osaki T, Komatsu A, et al (2002) Experimental infection of germ-free mice with hyper-toxigenic enterohaemorrhagic Escherichia coli O157:H7, strain 6. J Med Microbiol 51: 336–343. 11926740 10.1099/0022-1317-51-4-336

[19] Sugatani J, Igarashi T, Munakata M, Komiyama Y, Takahashi H, et al (2000) Activation of coagulation in C57BL/6 mice given verotoxin 2 (VT2) and the effect of co-administration of LPS with VT2. Thromb Res 100: 61–72. 11053618 10.1016/s0049-3848(00)00305-4

[20] Obata F, Tohyama K, Bonev AD, Kolling GL, Keepers TR, et al (2008) Shiga toxin 2 affects the central nervous system through receptor globotriaosylceramide localized to neurons. J Infect Dis 198: 1398–1406. 18754742 10.1086/591911 PMC2684825

[21] Nishikawa K, Matsuoka K, Kita E, Okabe N, Mizuguchi M, et al (2002) A therapeutic agent with oriented carbohydrates for treatment of infections by Shiga toxin-producing Escherichia coli O157:H7. Proc Natl Acad Sci USA 99: 7669–7674. 12032341 10.1073/pnas.112058999 PMC124317

[22] Okuda T, Tokuda N, Numata S, Ito M, Ohta M, et al (2006) Targeted disruption of Gb3/CD77 synthase gene resulted in the complete deletion of globo-series glycosphingolipids and loss of sensitivity to verotoxins. J Biol Chem 281: 10230–10235. 16476743 10.1074/jbc.M600057200

[23] Kita E, Yunou Y, Kurioka T, Harada H, Yoshikawa S, et al (2000) Pathogenic mechanism of mouse brain damage caused by oral infection with Shiga toxin-producing Escherichia coli O157:H7. Infect Immun 68: 1207–1214. 10678928 10.1128/iai.68.3.1207-1214.2000 PMC97269

[24] Watanabe M, Matsuoka K, Kita E, Igai K, Higashi N, et al (2004) Oral therapeutic agents with highly clustered globotriose for treatment of Shiga toxigenic Escherichia coli infections. J Infect Dis 189: 360–368. 14745692 10.1086/381124

[25] Boccoli J, Loidl CF, Lopez-Costa JJ, Creydt VP, Ibarra C, et al (2008) Intracerebroventricular administration of Shiga toxin type 2 altered the expression levels of neuronal nitric oxide synthase and glial fibrillary acidic protein in rat brains. Brain Res 1230: 320–333. 18675791 10.1016/j.brainres.2008.07.052

[26] Goldstein J, Loidl CF, Creydt VP, Boccoli J, Ibarra C (2007) Intracerebroventricular administration of Shiga toxin type 2 induces striatal neuronal death and glial alterations: an ultrastructural study. Brain Res 1161: 106–115. 17610852 10.1016/j.brainres.2007.05.067

[27] Bast DJ, Brunton JL, Karmali MA, Richardson SE (1997) Toxicity and immunogenicity of a verotoxin 1 mutant with reduced globotriaosylceramide receptor binding in rabbits. Infect Immun 65: 2019–2028. 9169727 10.1128/iai.65.6.2019-2028.1997 PMC175279

[28] Richardson SE, Rotman TA, Jay V, Smith CR, Becker LE, et al (1992) Experimental verocytotoxemia in rabbits. Infect Immun 60: 4154–4167. 1398926 10.1128/iai.60.10.4154-4167.1992 PMC257448

[29] Zoja C, Corna D, Farina C, Sacchi G, Lingwood C, et al (1992) Verotoxin glycolipid receptors determine the localization of microangiopathic process in rabbits given verotoxin-1. J Lab Clin Med 120: 229–238. 1323633

[30] Fujii J, Kinoshita Y, Kita T, Higure A, Takeda T, et al (1996) Magnetic resonance imaging and histopathological study of brain lesions in rabbits given intravenous verotoxin 2. Infect Immun 64: 5053–5060. 8945546 10.1128/iai.64.12.5053-5060.1996 PMC174488

[31] Mizuguchi M, Sugatani J, Maeda T, Momoi T, Arima K, et al (2001) Cerebrovascular damage in young rabbits after intravenous administration of Shiga toxin 2. Acta Neuropathol (Berl) 102: 306–312. 11603804 10.1007/s004010100384

[32] Takahashi K, Funata N, Ikuta F, Sato S (2008) Neuronal apoptosis and inflammatory responses in the central nervous system of a rabbit treated with Shiga toxin-2. J Neuroinflammation 5: 11. 18355415 10.1186/1742-2094-5-11 PMC2330034

[33] Matise I, Sirinarumitr T, Bosworth BT, Moon HW (2000) Vascular ultrastructure and DNA fragmentation in swine infected with Shiga toxin-producing Escherichia coli. Vet Pathol 37: 318–327. 10896393 10.1354/vp.37-4-318

[34] Dean-Nystrom EA, Pohlenz JF, Moon HW, O'Brien AD (2000) Escherichia coli O157:H7 causes more-severe systemic disease in suckling piglets than in colostrum deprived neonatal piglets. Infect Immun 68: 2356–2358. 10722643 10.1128/iai.68.4.2356-2358.2000 PMC97427

[35] Donohue-Rolfe A, Kondova I, Oswald S, Hutto D, Tzipori S (2000) Escherichia coli 0157:H7 strains that express Shiga toxin (Stx) 2 alone are more neurotropic for gnotobiotic piglets than are isotypes producing only Stx1 or both Stx1 and Stx2. J Infect Dis 181: 1825–1829. 10823794 10.1086/315421

[36] Tzipori S, Chow CW, Powell HR (1988) Cerebral infection with Escherichia coli O157:H7 in humans and gnotobiotic piglets. J ClinPathol 41: 1099–1103. 10.1136/jcp.41.10.1099 PMC1141695 3056980

[37] Tzipori S, Gunzer F, Donnenberg MS, de Montigny L, Kaper JB, et al (1995) The role of the eaeA gene in diarrhea and neurological complications in a gnotobiotic piglet model of enterohemorrhagic Escherichia coli infection. Infect Immun 63: 3621–3627. 7642299 10.1128/iai.63.9.3621-3627.1995 PMC173502

[38] Siegler RL, Pysher TJ, Tesh VL, Taylor FB Jr (2001) Response to single and divided doses of Shiga toxin-1 in a primate model of hemolytic uremic syndrome. J Am Soc Nephrol 12: 1458–1467. 11423574 10.1681/ASN.V1271458

[39] Taylor FB Jr, Tesh VL, DeBault L, Li A, Chang AC, et al (1999) Characterization of the baboon responses to Shiga-like toxin: descriptive study of a new primate model of toxic responses to Stx-1. Am. J Pathol 154: 1285–1299. 10.1016/S0002-9440(10)65380-1 PMC1866558 10233866

[40] Obata F (2010) Influence of Escherichia coli shiga toxin on the mammalian central nervous system. Adv Appl Microbiol 71: 1–19. 20378049 10.1016/S0065-2164(10)71001-7

[41] DiMario FJ Jr, Bronte-Stewart H, Sherbotie J, Turner ME (1987) Lacunar infarction of the basal ganglia as a complication of hemolytic-uremic syndrome. MRI and clinical correlations. Clin Pediatr (Phila) 26: 586–590. 3665330 10.1177/000992288702601106

[42] Jeong YK, Kim IO, Kim WS, Hwang YS, Choi Y, et al (1994) Hemolytic uremic syndrome: MR findings of CNS complications. Pediatr Radiol 24: 585–586. 7724282 10.1007/BF02012739

[43] Signorini E, Lucchi S, Mastrangelo M, Rapuzzi S, Edefonti A, et al (2000) Central nervous system involvement in a child with hemolytic uremic syndrome. Pediatr Nephrol 14: 990–992. 10975313 10.1007/s004670050059

[44] Chesselet MF, Delfs JM (1996) Basal ganglia and movement disorders: an update. Trends Neurosci 19: 417–422. 8888518 10.1016/0166-2236(96)10052-7

[45] Wichmann T, DeLong MR (1996) Functional and pathophysiological models of the basal ganglia. Curr Opin Neurobiol 6: 751–758. 9000030 10.1016/s0959-4388(96)80024-9

[46] Hager A, Staudt M, Klare B, von Einsiedel HG, Krägeloh-Mann I (1999) Hemolytic-remic syndrome with involvement of basal ganglia and cerebellum. Neuropediatrics 30: 210–3. 10569213 10.1055/s-2007-973492

[47] Barnett NDP, Kaplan AM, Bernes SM, Cohen ML (1995) Hemolytic uremic syndrome with particular involvement of basal ganglia and favorable outcome. Pediatr Neurol 12: 155–158. 7779215 10.1016/0887-8994(94)00121-h

[48] Steinborn M, Leiz S, Rudisser K, Griebel M, Harder T, et al (2004) CT and MRI in haemolyticuraemic syndrome with central nervous system involvement: distribution of lesions and prognostic value of imaging findings. Pediatr Radiol 34: 805–810. 15378218 10.1007/s00247-004-1289-2

[49] Candiano G, Bruschi M, Musante L, Santucci L, Ghiggeri GM, et al (2004) Blue silver: a very sensitive colloidal Coomassie G-250 staining for proteome analysis. Electrophoresis 25: 1327–1333. 15174055 10.1002/elps.200305844

[50] Tironi-Farinati C, Loidl CF, Boccoli J, Parma Y, Fernandez-Miyakawa ME, et al (2010) Intracerebroventricular Shiga toxin 2 increases the expression of its receptor globotriaosylceramide and causes dendritic abnormalities. J Neuroimmunol 222: 48–61. 20347160 10.1016/j.jneuroim.2010.03.001

[51] Wood NI, Pallier PN, Wanderer J, Morton AJ (2007) Systemic administration of Congo red does not improve motor or cognitive function in R6/2 mice. Neurobiol Dis 25: 342–353. 17095235 10.1016/j.nbd.2006.09.015

[52] Rogers DC, Fisher EMC, Brown SDM, Peters J, Hunter AJ, et al (1997) Behavioral and functional analysis of mouse phenotype: SHIRPA, a proposed protocol for comprehensive phenotype assessment. Mamm Genome 8: 711–713. 9321461 10.1007/s003359900551

[53] Priestley JV, Alvarez FJ, Averill S (1992) Pre-embedding electron microscopic immunocytochemistry. In: Polak, J.M., Priestley, J.V. (Eds.), Electron Microscopic Immunocytochemistry. Oxford University Press, Oxford, pp. 89–121.

[54] Northington FJ, Zelaya ME, O'Riordan DP, Blomgren K, Flock DL, et al (2007) Failure to complete apoptosis following neonatal hypoxia-ischemia manifests as "continuum" phenotype of cell death and occurs with multiple manifestations of mitochondrial dysfunction in rodent forebrain. Neuroscience 149: 822–33. 17961929 10.1016/j.neuroscience.2007.06.060 PMC3947608

[55] Ruan YW, Ling GY, Zhang JL, Xu ZC (2003) Apoptosis in the adult striatum after transient forebrain ischemia and the effects of ischemic severity. Brain Res 982: 228–240. 12915258 10.1016/s0006-8993(03)03021-x

[56] Petito CK, Pulsinelli WA, Jacobson G, Plum F (1982) Edema and vascular permeability in cerebral ischemia: comparison between ischemic neuronal damage and infarction. J Neuropathol Exp Neurol 41: 423–436. 7086465 10.1097/00005072-198207000-00005

[57] Grundmann K, Glöckle N, Martella G, Sciamanna G, Hauser TK, et al (2012) Generation of a novel rodent model for DYT1 dystonia. Neurobiol Dis 47: 61–74. 22472189 10.1016/j.nbd.2012.03.024

[58] Roos RAC, Bots GThAM (1983) Nuclear membrane indentations in Huntington's chorea. J Neurol Sci 61: 37–47. 6226764 10.1016/0022-510x(83)90053-9

[59] Bots GThAM, Bruyn GW (1987) Neuropathological changes of the nucleus accumbens in Huntington's chorea. Acta Neuropathol 55: 21–22. 10.1007/BF00691525 6215820

[60] Davies SW, Turmaine M, Cozens BA, DiFiglia M, Sharp AH, et al (1997) Formation of neuronal intranuclear inclusions underlies the neurological dysfunction in mice transgenic for the HD mutation. Cell 90: 537–548. 9267033 10.1016/s0092-8674(00)80513-9

[61] Lieberman AR (1971) A review of the principal features of perikaryal responses to axon injury. Int Rev Neurobiol 14: 49–124. 4948651 10.1016/s0074-7742(08)60183-x

[62] Malamud N, Hirano A (1974) Atlas of Neuropathology-2nd revised edition, Berkeley and Los Angeles: University of California Press. pp 36–38.

[63] Lei M, Hua X, Xiao M, Ding J, Han Q, et al (2008) Impairments of astrocytes are involved in the d-galactose-induced brain aging. Biochem Biophys Res Commun 369: 1082–1087. 18329384 10.1016/j.bbrc.2008.02.151

[64] Garbuzova-Davis S, Louis MK, Haller EM, Derasari HM, Rawls AE, et al (2011) Blood-Brain Barrier Impairment in an Animal Model of MPS III B. PLoS ONE 6(3): e16601 doi:10.1371/journal.pone.0016601. 10.1371/journal.pone.0016601 PMC3049764 21408219

[65] Ballabh P, Braun A, Nedergaard M (2004) The blood-brain barrier: an overview: structure, regulation, and clinical implications. Neurobiol Dis 16: 1–13. 15207256 10.1016/j.nbd.2003.12.016

[66] Moran LB, Graeber MB (2004) The facial nerve axotomy model. Brain Res Brain Res Rev 44: 154–178. 15003391 10.1016/j.brainresrev.2003.11.004

[67] Barron KD, Marciano FF, Amundson R, Mankes R (1990) Perineuronal glial responses after axotomy of central and peripheral axons. A comparison. Brain Res 523: 219–229. 1698104 10.1016/0006-8993(90)91490-8

[68] Pastor AM, Moreno-López B, De La Cruz RR, Delgado-García JM (1997) Effects of botulinum neurotoxin type A on abducens motoneurons in the cat: ultrastructural and synaptic alterations. Neuroscience 81: 457–478. 9300434 10.1016/s0306-4522(97)00200-5

[69] Murata F, Spicer SS (1974) Ultrastructural comparison of basophilic leukocytes and mast cell in the guinea pig. Am J Anat 139: 335–352.

[70] Florenzano F, Bentivoglio M (2000) Degranulation, density and distribution of mast cells in the rat thalamus: a light and electron microscopic study in basal conditions and after intracerebroventricular administration of nerve growth factor. J Comp Neurol 424: 651–669. 10931487

[71] Strbian D, Karjalainen-Lindsberg ML, Tatlisumak T, Lindsberg PJ (2006) Cerebral mast cells regulate early ischemic brain swelling and neutrophil accumulation. J Cereb Blood Flow Metab 26: 605–612. 16163296 10.1038/sj.jcbfm.9600228

[72] Uranova N, Orlovskaya D, Vikhreva O, Zimina I, Kolomeets N, et al (2001) Electron microscopy of oligodendroglia in severe mental illness. Brain Res Bull 55: 597–610. 11576756 10.1016/s0361-9230(01)00528-7

[73] Kohutnicka M, Lewandowska E, Kurkowska-Jastrzebska I, Członkowski A, Członkowska A (1998) Microglial and astrocytic involvement in a murine model of Parkinson's disease induced by 1-methyl-4-phenyl-1,2,3,6-tetrahydropyridine (MPTP). Immunopharmacology 39: 167–180. 9754903 10.1016/s0162-3109(98)00022-8

[74] Berciano MT, Fernandez R, Pena E, Calle E, Villagra NT, et al (1999) Necrosis of schwann cells during tellurium-induced primary demyelination: DNA fragmentation, reorganization of splicing machinery, and formation of intranuclear rods of actin. J Neuropathol ExpNeurol 58: 1234–1243. 10.1097/00005072-199912000-00004 10604748

[75] Pardridge WM (1999) Blood-brain barrier biology and methodology. J NeuroVirol 5: 556–569. 10602397 10.3109/13550289909021285

[76] Trachtman H, Austin C, Lewinski M, Stahl RA (2012) Renal and neurological involvement in typical Shiga toxin-associated HUS. Nat Rev Nephrol 11: 658–669. 10.1038/nrneph.2012.196 22986362

[77] Louise CB, Obrig TG (1991) Shiga toxin-associated hemolytic-uremic syndrome: combined cytotoxic effects of Shiga toxin, interleukin-1 beta, and tumor necrosis factor alpha on human vascular endothelial cells in vitro. Infect Immun 59: 4173–4179. 1937774 10.1128/iai.59.11.4173-4179.1991 PMC259013

[78] Burdet J, Sacerdoti F, Cella M, Franchi AM, Ibarra C (2012) Role of TNF-α in the Mechanisms Responsible for Preterm Delivery Induced by Stx2 in Rats. Br J Pharmacol doi:10.1111/j.1476-5381.2012.02239.x. 10.1111/j.1476-5381.2012.02239.x PMC3631382 23043728

[79] Dimagl U, Iadecola C, Moskowitz MA (1999) Pathobiology of ischaemic stroke: an integrated view. TINS 22 (9): 391–397. 10.1016/s0166-2236(99)01401-0 10441299

[80] Gerfen CR (2004) Basal ganglia. In: Paxinos, G. (Ed.), The Rat Nervous System, third edition.Elsevier Academic Press, London, pp. 455–508.

[81] West MO, Carelli RM, Pomerantz M, Cohen SM, Gardner JP, et al (1990) A region in the dorsolateral striatum of the rat exhibiting single-unit correlations with specific locomotor limb movements. J Neurophysiol 64: 1233–1246. 2258744 10.1152/jn.1990.64.4.1233

